# A Mobile App to Rapidly Appraise the In-Store Food Environment: Reliability, Utility, and Construct Validity Study

**DOI:** 10.2196/16971

**Published:** 2020-07-22

**Authors:** Emma Joy McMahon, Rachael Jaenke, Julie Brimblecombe

**Affiliations:** 1 Wellbeing and Preventable Chronic Disease Division Menzies School of Health Research Charles Darwin University Darwin Australia; 2 Department of Nutrition, Dietetics and Food Faculty of Medicine, Nursing and Health Sciences Monash University Melbourne Australia

**Keywords:** mobile apps, reliability and validity, food, diet, environment and public health

## Abstract

**Background:**

Consumer food environments are increasingly being recognized as influential determinants of food purchasing and subsequent intake and health. We developed a tool to enable efficient, but relatively comprehensive, appraisal of the in-store food environment. The Store Scout mobile app facilitates the evaluation of product (availability and range), placement (visibility, accessibility, proximity to high-traffic areas, and location relative to other products), price (price promotion), and promotion (displays and advertising) across 7 categories of food products, with appraisal given immediately as scores (0-100, where a higher score is more in line with best practice). Primary end users are public health nutritionists and nutritionists employed by store organizations; however, store managers and staff are also potential end users.

**Objective:**

This study aims to evaluate the reliability (interrater reliability and internal consistency), utility (distribution of scores), and construct validity (score by store type) of measurements using the Store Scout mobile app.

**Methods:**

The Store Scout mobile app was used independently by 2 surveyors to evaluate the store environment in 54 stores: 34 metropolitan stores (9 small and 11 large supermarkets, 10 convenience stores, and 4 petrol stations) in Brisbane, Australia, and 20 remote stores (19 small supermarkets and 1 petrol station) in Indigenous Australian communities in Northern Australia. The agreement between surveyors in the overall and category scores was evaluated using intraclass correlation coefficients (ICCs). Interrater reliability of measurement items was assessed using percentage agreement and the Gwet agreement coefficient (AC). Internal consistency was assessed by comparing the responses of items measuring similar aspects of the store environment. We examined the distribution of score values using boxplots and differences by store type using the Kruskal-Wallis test.

**Results:**

The median difference in the overall score between surveyors was 4.4 (range 0.0-11.1), with an ICC of 0.954 (95% CI 0.914-0.975). Most measurement items had very good (n=74/196, 37.8%) or good (n=81/196, 41.3%) interrater reliability using the Gwet AC. A minimal inconsistency of measurement was found. Overall scores ranged from 19.2 to 81.6. There was a significant difference in score by store type (*P*<.001). Large Brisbane supermarkets scored highest (median 77.4, range 53.2-81.6), whereas small Brisbane supermarkets (median 63.9, range 41.0-71.3) and small remote supermarkets (median 63.8, range 56.5-74.9) scored significantly higher than Brisbane petrol stations (median 33.1, range 19.2-37.8) and convenience stores (median 39.0, range 22.4-63.8).

**Conclusions:**

These findings suggest good reliability and internal consistency of food environment measurements using the Store Scout mobile app. We identified specific aspects that can be improved to further increase the reliability of this tool. We found a good distribution of score values and evidence that scoring could capture differences by store type in line with previous evidence, which gives an indication of construct validity. The Store Scout mobile app shows promise in its capability to measure and track the health-enabling characteristics of store environments.

## Introduction

### Background

An unhealthy diet is one of the leading risk factors for noncommunicable diseases, which are now the leading cause of preventable death worldwide [1]. Consumer food retail environments are increasingly being recognized as influential determinants of food purchasing, diet quality, and health outcomes [2-6]. They not only determine the type of foods consumers can access and the convenience and the cost of accessing them but can also influence the appeal of these foods [1]. Health-enabling food environments are those that make healthy diets available, affordable, accessible, and appealing [1].

We developed a mobile app, Store Scout, to support rapid appraisal of the in-store food environment, drawing from a review of existing tools for assessing the healthiness of food environments, evidence for best practice (for healthy food supply or diet), stakeholder consultation, and pilot testing [7]. This tool was initially developed as a paper-based instrument for use in remote stores in Australian Aboriginal and Torres Strait Islander communities [7]. We found the paper version of the tool to be suitable for use outside this setting when pilot testing in nonremote stores in Brisbane (the capital city of Queensland, Australia) and Darwin (the capital city of the Northern Territory, Australia). During pilot testing, we found that retailers in both remote and nonremote samples were frequently interested in feedback on the results of their store. The current mobile app version of this tool enables immediate appraisal of the store environment against best practice (as scores), which may be an effective mechanism for communicating with decision makers to support the implementation and monitoring of health-enabling food environments [7]. Primary end users are public health nutritionists and nutritionists employed by store organizations; however, store managers and staff also identified as potential end users during stakeholder consultation [7].

### Objectives

This study aims to evaluate interrater reliability (of measurement items and scores), internal consistency, utility of scoring, and construct validity (scores by type of store) of measurements taken using the Store Scout app. Establishing the interrater reliability of store environment measurements is important to ensure consistency of measurements between different raters [8]. Measurement of internal consistency can also be used to assess the reliability of a measurement; however, it is reported for only 3.5% of studies evaluating store environment measurements using psychometric measures (whereas 16.7% report interrater reliability) [3]. Examining the distribution of scores and variation by store type will be useful in exploring the utility of the scoring system in distinguishing between store environments. Furthermore, we can examine if differences by store type are in line with other evidence as an assessment of construct validity (the extent to which the measure is related to other constructs in an expected way [3]).

## Methods

### Study Sample

The Store Scout app was used to evaluate the store environment in 2 samples: (1) 20 remote indigenous community stores in the Northern Territory and Northern Queensland, Australia, as part of the Healthy Stores 2020 study (ACTRN12618001588280) [9]; and (2) 34 metropolitan stores in Brisbane, the capital city of Queensland, Australia (including supermarkets, convenience stores, and petrol stations), prioritizing those in the most disadvantaged areas of Brisbane for better comparability with the remote sample. The Australian Bureau of Statistics 2016 Index of Relative Socio-economic Advantage and Disadvantage (IRSAD), an ordinal index score derived from social and economic variables from the 2016 national Australian census (eg, income, employment status, education level, disability and health conditions, and household overcrowding [10]), was used to identify Brisbane suburbs (statistical area level 2) with the highest levels of disadvantage (lowest IRSAD score [11]).

Approximately 19% of Aboriginal and Torres Strait Islander peoples live in remote or very remote areas of Australia in small towns, commonly referred to as communities and/or homelands [12]. These communities vary in size, with most having less than 1000 people. In remote and very remote Aboriginal and Torres Strait Islander communities, most food is sourced from the local community retail store [13]. These stores are small- to medium-sized retail businesses that sell a wide range of food products as well as other household items, and therefore, they can be classified as (small or medium) supermarkets. The Healthy Store 2020 study was conducted in collaboration with the Arnhem Land Progress Aboriginal Corporation (ALPA), one of the largest remote retail store organizations and employers of Aboriginal and Torres Strait Islander people in Australia. This study included 20 stores in remote communities in the Northern Territory and in the Torres Strait Island and Cape York regions of far north Queensland. These communities had a median population of 740 (range 220-2560), and a median 93% of the population identified as Aboriginal or Torres Strait Islander (range 69%-97%) [12]. Further details of this study, which examined the effectiveness of a merchandising strategy to reduce discretionary food purchasing, are available in the study protocol [9].

Brisbane is the capital city of Queensland, Australia, and the third most populous city in Australia. We aimed for a sample of 30 to 50 Brisbane food stores. Food stores were identified in each suburb using web-based maps and business listings, beginning with the most disadvantaged suburb (lowest ISRAD score [11]) and continuing through the suburbs (in order of ascending ISRAD score) until at least 50 stores that appeared to meet the inclusion criteria were identified. The inclusion criteria were Brisbane stores likely to sell foods from all the five food groups as defined by the Australian dietary guidelines (fruit; vegetables; dairy; meat, fish and eggs; and breads and cereals [14]) and a range of both ready-to-eat and non–ready-to-eat food items (including supermarkets, convenience stores, and some petrol stations and excluding cafes and restaurants that usually sell only ready-to-eat items). Exclusion criteria were stores that were not easily accessible by car (required boat access and >1-hour travel time from central Brisbane), in areas surveyed during paper tool development (South Brisbane areas), and specialty food stores.

Using these methods, 50 stores from 10 suburbs in North, East, and West Brisbane were identified. No stores were in the inner-city suburbs (because of these having lower IRSAD scores; ie, less disadvantage). All 50 stores were visited, although 3 were found to be closed (permanently), 2 did not meet the inclusion criteria (1 store had been converted to a café and 1 store sold spices only), and in 1 store, surveyors reported that the app was not loading. Of the remaining 44 eligible stores, 34 provided verbal consent and 10 did not provide consent (no one available with the appropriate authority to consent or language barrier), giving a 77% participation rate.

Although the 34 Brisbane stores surveyed were in some of the most disadvantaged suburbs of Brisbane, these areas were less disadvantaged relative to the areas the remote stores were located. Of the 20 remote stores, 19 were in the first decile (most disadvantaged) areas of Australia, whereas most Brisbane stores were in the fifth (23/34) and sixth (9/34) deciles (as reported by the Australian Bureau of Statistics deciles of the 2016 ISRAD by statistical area level 2 [11]). The remaining 2 Brisbane stores were in the third and fourth deciles, whereas the remaining remote store was in the third decile [11].

### Data Collection

The Store Scout app includes measurement items across 7 sections, one for each of the 7 food and drink categories with questions related to product (availability and product range), placement (visibility, accessibility, proximity to high-traffic areas, and location relative to other products), price (price promotion), and promotion (displays, advertising, or activity) for types of products in each category, including healthier and less healthy options. Definitions related to product type or store environment elements are provided in the tooltips for each measurement item. Healthiness definitions used nutrient criteria (eg, >15 g fiber per 100 g) or descriptive terms (eg, lean meat) based on other nutrition resources or guidelines used in Australia [14-16]. There is an additional section of store manager questions and suggested strategies, but these do not affect scoring and were not collected in this study.

Store Scout scoring is calculated from up to 199 yes or no measurement items, each of which is worth 1 possible point. As 6 scoring measurement items are hidden if the response to a previous measurement item was *no*, there are up to 193 to 199 possible points across the 7 categories (breads and cereals: 20, dairy and eggs: 29, drinks: 28, fruits and vegetables: 30, meals and convenience foods: 24-30, meat and seafood: 21, and snack foods: 41). Practices related to both healthy products and unhealthy (or less healthy) products contribute to scoring. A point is awarded for a *yes* response to practices likely to encourage purchases of healthy products (eg *Fresh fruit at or near checkout*) or discourage purchases of unhealthy or less healthy products (eg *Limited shelf space for unhealthy products*) or for a *no* response for practices likely to encourage purchases of unhealthy (or less healthy) products (eg, *Lollies* [confectionery]*, chocolate & chips at or near checkouts*). Scores (range 0-100, a higher score indicates a more *health-enabling* store) are calculated for each category as a percentage of total possible points averaged across the 7 categories to give an overall score.

Data are transferred to a web-based portal once the user connects to the internet. The web-based portal was under development during this study, and there were intermittent issues with data loss during transfer. Where these issues were known ahead of time, screenshots of completed data screens were taken as backup, and data were entered manually; however, some data were missing despite this (Multimedia Appendix 1). Category scores and overall scores were calculated (as described earlier) only for complete data (ie, where there were no missing data).

A total of 68 surveys were conducted in 34 Brisbane food stores in April 2018. In each store, 2 surveyors with nutrition expertise (final year Bachelor of Nutrition and Dietetics students) who had no previous experience with the Store Scout tool before this project used the Store Scout app to simultaneously, but independently, measure the in-store environment. Data collection occurred in 2 stages. Initially, surveyors were given training only on the basics of how to use the app. After 17 stores had been surveyed (stage 1), the surveyors and 2 members of the research team discussed specific questions where agreement was lowest to inform training materials and identify where tooltips could be improved. This discussion mostly related to differences in inclusion or exclusion of products into the specific categories (especially for meals and convenience foods); classification of products as healthy or less healthy; definition of promotional materials; and definitions of key terms such as *high traffic areas*, *convenience meal*, and *limited range*. The 2 surveyors then surveyed the remaining 17 stores (stage 2). Surveyors were asked to count the number of registers as a surrogate for store size.

In the remote store sample, measurement of the store environment using the Store Scout app occurred between September 2018 and May 2019 at the end of each study period: baseline, intervention, and postintervention. Measurements were taken by a single surveyor except for at the end of the intervention period (November 2018 to December 2018), where 2 surveyors independently measured the store environment to enable the assessment of interrater reliability. Surveyors included members of the research team and public health nutritionists, most of whom had no previous experience in using the tool besides a short (approximately 30-60 min) training session.

This project was granted ethics approval by the Top End Human Research Ethics Committee (HREC 2017-2820 and HREC-2018-3048) and the Far North Queensland Human Research Ethics Committee (HREC-18-QCH-23-1211).

### Statistical Analysis

We assessed interrater reliability (of measurement items and scores), internal consistency, utility of scoring, and construct validity (scores by store type). The data included depended on the aim of the analysis and unit of analysis (Multimedia Appendix 2). All analyses were performed using Stata Statistical Software, version 16.0 (StataCorp) [17].

We assessed the interrater reliability of the 196 core (always shown) yes or no measurement items using percentage agreement and the Gwet agreement coefficient (AC) [18]. We also reported kappa statistics for comparability with other tools, although these are less suitable because of their dependence on the homogeneity of marginal distributions [8,19,20]. Coefficients were interpreted using the following cutoffs: slight or poor (<0.2), fair (0.21-0.4), moderate (0.41-0.6), good (0.61-0.8), and very good (0.81-1) [21].

We assessed the interrater reliability of scores (overall and by category) using intraclass correlation coefficients (ICCs) and median and range of the difference between scores (as the difference between scores was not normally distributed). The ICC model and estimates and their 95% CIs were calculated using a one-way random effects model based on an individual rater, as recommended in the guideline by Koo and Li [22] for reliability experiments where subjects are assessed using different sets of raters and the measurement will be taken by a single rater in usual practice (ie, outside of a reliability experiment). ICC values were interpreted using the following cutoffs: poor (<0.5), moderate (0.5-0.75), good (0.75-0.9), and excellent (>0.90) [22].

We did not examine interrater reliability by surveyor group or sample because of the reduced sample size and differential variance in these groups. Instead, we examined the level of agreement between surveyors overall (combined sample) and in each surveyor group or level of training (Brisbane stage 1, Brisbane stage 2, and remote stores) as percentage agreement across all measurement items and median and range of the difference between scores (as the difference between scores was not normally distributed).

Internal consistency was assessed on all surveys collected by comparing responses to related measurement items, classified as higher-order items (eg, “Is there promotional material or activity for healthier breads and cereals?”) and more specific follow-up items (eg, “Does the promotion stand out?”). Surveys where responses to both measurement items were *yes* or *no* were considered internally consistent. Surveys where there was a *no* response to the higher-order measurement item but a *yes* response to the more specific follow-up item were inconsistent. Cases where there was a *yes* response to the higher-order item and a *no* response to the more specific item were excluded (as it was not possible to assess the consistency of these). Internal consistency was calculated as the percentage of surveys where responses were consistent.

We examined the extent to which Store Scout scores resulted in a distribution of values using boxplots (of scores where an average of the 2 values was used if collected by more than one surveyor).

Difference by store type was evaluated using the Kruskal-Wallis equality-of-populations rank test with Dunn pairwise comparison of scores and correction for multiple comparisons (Holm-Bonferroni). It was expected that petrol stations and convenience stores would score lower than supermarkets [23].

## Results

### Store Characteristics

Table 1 shows the types of stores surveyed in each sample. A cutoff of ≤5 registers was used to classify Brisbane supermarkets as small or large to enable comparison with remote stores, which had 1-4 registers in each store. The remote store that functioned primarily as a petrol station was excluded from the analysis of score by store type.

**Table 1:** Store characteristics.

Store type			n	Number of registers^a^, median (range)	Time taken to survey (min), median (range)
**Brisbane stores**					
	All Brisbane stores		34	3 (1-14)	25 (10-40)
	Petrol stations		4	2 (1-3)	15 (10-20)
	Convenience stores		10	1 (1-3)	15 (10-30)
	**Supermarkets**				
		Both small and large supermarkets	20	6 (2-14)	30 (15-40)
		Small supermarkets	9	4 (2-5)	30 (15-30)
		Large supermarkets	11	9 (6-14)	30 (20-40)
**Remote stores**					
	Both remote supermarkets and petrol stations		20	2.5 (1-4)	—^b^
	Remote supermarkets		19	3 (1-4)	—
	Petrol stations		1	2	—

^a^Excludes self-serve registers (only seen in large stores).

^b^Time taken to complete the surveys was not collected in the remote sample.

### Interrater Reliability

Most measurement items had >80% agreement between surveyors (n=129/196, 65.8%) and very good (n=74/196, 37.8%) or good (n=81/196, 41.3%) interrater reliability using the Gwet AC (Table 2). There were 3 measurement items that had slight or poor interrater reliability; these combined a judgment of healthiness (healthier or less healthy snack or healthier meals and convenience foods) as well as a subjective judgment of placement or range (*near checkout*, *limited range*, and *same or more space*). Using kappa coefficients, most had moderate (n=72/196, 36.7%) or good or very good (n=72/196, 36.7%) interrater reliability. Of the 18 items with slight or poor interrater reliability assessed by kappa coefficients, most surveyors had >70% (n=11/18) agreement, and some surveyors had >80% (n=5/18) agreement.

Overall, the scores had excellent interrater reliability, as indicated by ICC (0.954; 95% CI 0.914-0.975). The ICC for category scores indicated excellent interrater reliability for fruits and vegetables (0.940; 95% CI 0.898-0.966); good interrater reliability for dairy and eggs (0.881; 95% CI 0.798-0.932), meat and seafood (0.875; 95% CI 0.791-0.926), drinks (0.799; 95% CI 0.671-0.881), snack foods (0.784; 95% CI 0.648-0.872), and breads and cereals (0.727; 95% CI 0.568-0.834); and moderate interrater reliability for meals and convenience foods (0.696; 95% CI 0.517-0.817).

Overall, there was 83.20% (8584/10312) agreement across all measurement items collected by 2 surveyors in the combined sample. The percentage agreement increased from Brisbane stage 1 (n=2634/3278, 80.35%) to stage 2 (n=2764/3176, 87.00%), with the greatest increase within a category being in meal and convenience foods, likely because of clarification of which products fell into this category. Agreement was 83.6% in remote stores, with all categories having >80% agreement (Multimedia Appendix 3).

The median difference in overall scores between surveyors was 4.4 in the combined sample (range 0.0-11.1), with the smallest median difference in the remote sample (Table 3). There were larger differences between surveyors in category scores than overall scores.

**Table 2:** Store Scout measurement items by interrater reliability category (N=196).

Category	Agreement, n (%)	Gwet agreement coefficient, n (%)	Value, n (%)
Slight or poor (<0.2)	0 (0)	3 (2)	18 (9)
Fair (0.21-0.4)	0 (0)	6 (3)	34 (17)
Moderate (0.41-0.6)	3 (2)	32 (16)	72 (37)
Good (0.61-0.8)	64 (33)	81 (41)	54 (28)
Very good (0.81-1)	129 (66)	74 (38)	18 (9)

**Table 3:** Score difference between surveyors by category and sample.

Score^a^	All		Brisbane stage 1		Brisbane stage 2		Healthy stores 2020	
	n	Median (range)	n	Median (range)	n	Median (range)	n	Median (range)
Overall	39	4.4 (0.0-11.1)	13	5.5 (0.4-11.1)	15	5.3 (0.7-8.7)	11	2.2 (0.0-6.9)
Breads and cereals	52	5.0 (0.0-45.0)	16	10.0 (0.0-45.0)	16	5.0 (0.0-15.0)	19	5.0 (0.0-30.0)
Dairy and eggs	47	6.9 (0.0-27.6)	15	6.9 (0.0-20.7)	15	3.5 (0.0-27.6)	17	6.9 (0.0-17.3)
Drinks	49	7.1 (0.0-25.0)	17	7.1 (0.0-25.0)	16	9.0 (0.0-25.0)	16	3.6 (0.0-17.9)
Fruits and vegetables	51	6.6 (0.0-20.0)	17	6.6 (0.0-16.7)	16	3.4 (0.0-20.0)	18	6.7 (0.0-20.0)
Meals and convenience	48	12.5 (0.0-41.7)	16	19.1 (0.0-41.7)	16	4.2 (0.0-24.1)	16	8.4 (0.0-33.4)
Meat and seafood	51	9.5 (0.0-38.1)	17	9.5 (0.0-28.6)	15	4.8 (0.0-38.1)	19	9.5 (0.0-23.8)
Snack foods	49	4.9 (0.0-39.0)	16	4.9 (0.0-21.9)	16	6.1 (0.0-39.0)	17	2.5 (0.0-21.9)

^a^Scores were calculated only for complete data (ie, where there were no missing data in the category).

### Internal Consistency

There was a small amount of inconsistency with some measurement items. In Brisbane stage 1 (n=34), all measurement items had >89% consistency, and 57% (n=20/35) of measurement items had perfect (100%) internal consistency. In Brisbane stage 2 (n=33 surveys), all measurement items had >95% consistency, and 76% (n=27/35) of measurement items had perfect internal consistency. In the remote sample (n=79 surveys), all measurement items had >98% consistency, and 74% (n=26/35) of measurement items had perfect internal consistency. Multimedia Appendix 4 shows the internal consistency percentage for each measurement item by sample.

### Utility of Scoring

**Figure 1:**
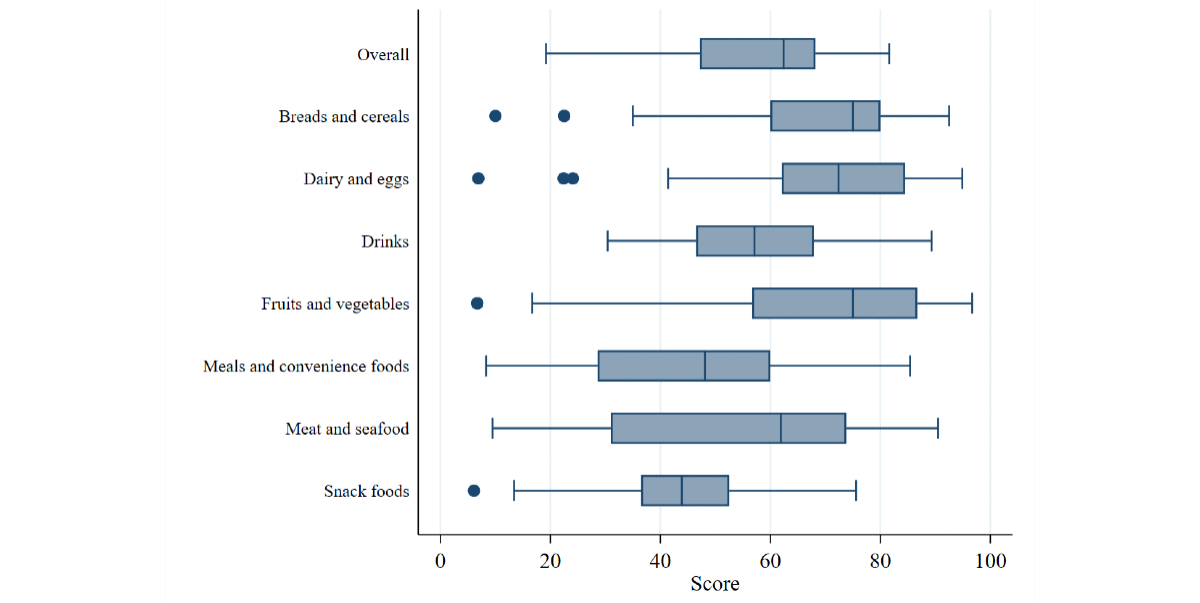
Distribution of overall and category scores for the combined sample of 54 stores.

Figure 1 shows the distribution of overall and category scores in the 54 stores. Overall scores ranged from 19.2 to 81.6, and there was acceptable variation within each category.

### Construct Validity

**Figure 2:**
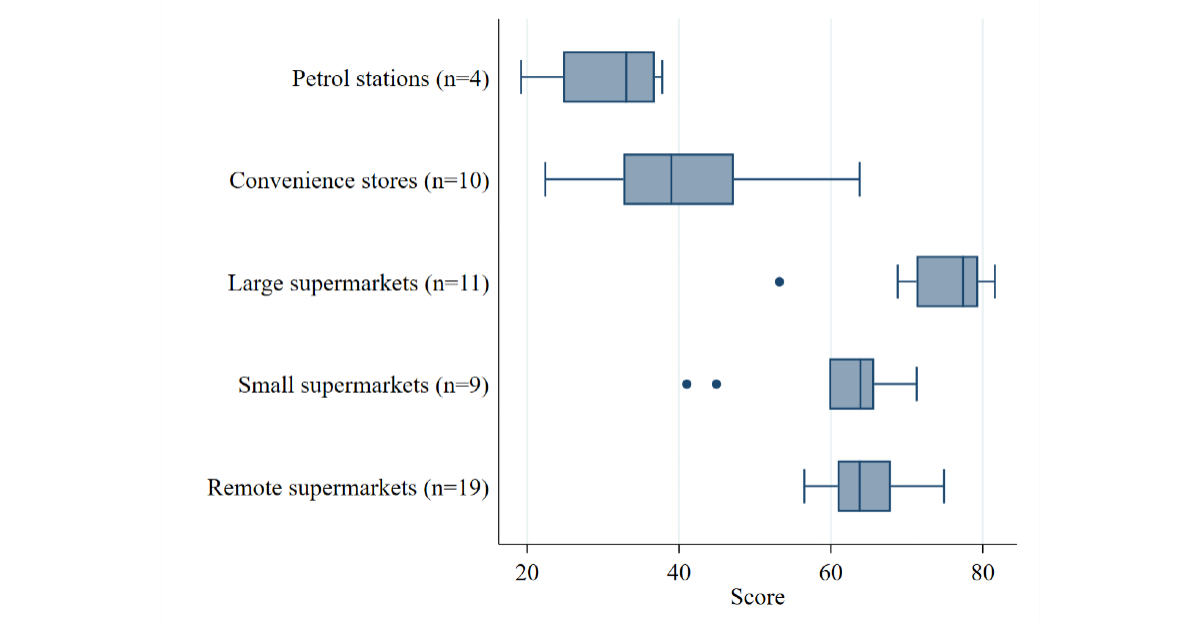
Distribution of overall scores by store type.

Figure 2 shows the distribution of scores by store type. There was a significant difference in scores by store type (*P*<.001 using the Kruskal-Wallis test), with large Brisbane supermarkets scoring significantly higher (median 77.4, range 53.2-81.6) than all other types of stores, and small Brisbane supermarkets (median 63.9, range 41.0-71.3) and remote supermarkets (median 63.8, range 56.5-74.9) scoring significantly higher than Brisbane convenience stores (median 39.0, range 22.4-63.8) and Brisbane petrol stations (median 33.1, range 19.2-37.8). After adjustment for multiple comparisons using the Holm-Bonferroni method, the difference between small Brisbane supermarkets and Brisbane convenience stores or petrol stations was no longer significant (Multimedia Appendix 5).

Multimedia Appendix 6 shows the distribution of points from measurement items related to healthy products (n=156 measurement items) or unhealthy products (n=43 measurement items) by store type. Although large supermarkets had the highest points for measurement items related to healthy products (median 136, range 73-140), they scored lowest (median 14, range 9-33) after petrol stations (median 12, range 11-14) for measurement items related to unhealthy products (least in line with best practice because of more availability, prominent placement, promotion, and/or pricing promotion of unhealthy products). Remote supermarkets scored highest (median 21, range 15-29) for measurement items related to unhealthy products (most in line with best practice because of less availability, prominent placement, promotion, and/or pricing promotion of unhealthy products).

## Discussion

### Principal Findings

We found good to very good interrater reliability of most measurement items, high agreement between scores, and high internal consistency of measurements when the in-store food environment was evaluated using the Store Scout app. We found good variation in scores across the samples, which include a variety of types of stores, including petrol stations, convenience stores, supermarkets, and remote stores, and evidence that the scoring system could capture differences by type of store in line with previous evidence, providing evidence of construct validity.

We found excellent interrater reliability of overall scores and good to excellent interrater reliability for all category scores, except meals and convenience foods scores, which had moderate interrater reliability. Although the median difference between surveyors in the overall score was acceptable (4.4), the median difference for category scores was larger. This is because of the number of measurement items contributing to category scores (20-41 depending on category) versus overall score (193-199), meaning that a difference between surveyors in one measurement item would mean a 2.4%-5% difference in category scores but a 0.3%-0.7% difference in the overall score. This means that overall scores are more reliable than category scores when looking at change (within store) or difference (between stores) with different surveyors.

There were 3 measurement items with slight or poor interrater reliability, as assessed by the Gwet AC. These measurement items required the surveyor to make judgments about placement or product range of healthier or less healthy products. It is likely that the reliability of some measurement items could be improved by more specific guiding definitions; for example, *at or near checkout* may be better defined by a perimeter that would be considered near, and *limited range* with a guideline of how many products or product facings this would be. Assessing if there is greater shelf space dedicated to healthier versus less healthy products is probably one of the more difficult items for surveyors, especially as it often requires assessing the healthfulness of every item in the category, sometimes needing to check the nutritional information, and it can be difficult to estimate space allocation when products are often found in numerous areas of a store. In this case, it may be better to instruct surveyors to focus on the primary area where the product is located. It is likely that the interrater reliability of measurement items will be strengthened by minimizing the subjectivity of measurement items.

We found perfect internal consistency (no identified inconsistency) for most measurement items where this could be assessed. Measurement items where a small degree of inconsistency was observed tended to have less specific wording regarding the product type that was assessed. These mostly had wording such as *healthier option* rather than the specific product type (which is listed in a header above the measurement items) and were examining a specific product type within the higher-order food group category. For example, the *healthier option easy to find* was related to healthier sweet biscuits and cakes, but the surveyor may have answered this more broadly (for any healthier option in the snack foods category). Modifying this to *healthier sweet biscuits or cakes easy to find* may make this clearer.

We found an increase in agreement and internal consistency from Brisbane stage 1 to Brisbane stage 2. In Brisbane stage 1, surveyors had no specific training or preexisting familiarity with the tool and did not have previous experience in retail food environment research but still achieved an 80% agreement rate. After discussing some key definitions (stage 2), this was increased to 87%. These discussions led to improvements in definitions in the app to reduce ambiguity (mostly related to categorization of products in categories and as healthy or unhealthy options), and learning was incorporated into the training of surveyors before data collection in the remote stores. Surveyors in the remote store sample were similar to those who would use the tool in practice in terms of having experience in retail food environment research and/or practice. Most had either not used the Store Scout tool before or had only done so once or twice, and most received only 30-60 min of training. Despite this, surveyors agreed 83% of the time, and the median difference in overall scores was only 2.2 (range 0-6.9).

Stores servicing smaller populations have reduced opportunities for economies of scale. Remote stores have additional logistical barriers to providing high-quality fresh food because of the long distance the food travels to get to the store [24,25]. High outside temperatures and poor road conditions (or road closures) can contribute to this. Therefore, remote stores have food demand and supply constraints that determine the price, quality, and variety of healthy foods that they can offer. Despite these challenges, we found that the store environment, as assessed by the Store Scout app in the 19 small remote supermarkets, scored similarly to small supermarkets in Brisbane and higher than convenience stores. When only the health-enabling practices related to unhealthy or less healthy products were considered (eg, not stocking or promoting unhealthy products or not placing unhealthy products in high-traffic areas), larger supermarkets and petrol stations scored the least points, whereas the small remote supermarkets scored the most. This is an encouraging finding; there has been considerable work aimed at improving food supply and access in the remote Australian Aboriginal and Torres Strait Islander communities where these stores are located [26-30], which has likely contributed to healthier food environments. ALPA (the organization overseeing the management of these remote stores) has demonstrated long-term commitment to promoting health and nutrition in the communities it serves, as demonstrated by their nutrition policy (first implemented in the early the 1980s), which includes strategies to increase the availability and affordability of nutritious foods [30].

### Comparison With Prior Work

We included kappa values to enable comparison with evaluations of other tools and found kappa coefficients that compare reasonably well with other tool evaluations, which generally report coefficients of around 0.7 [3]. Notably, other studies that have used kappa values could not include all measurement items because of the high and/or low prevalence of store traits measured [31,32]. Overall, 2 instances of excellent interrater reliability include that found for the Nutrition Environment Measures Survey in 88 stores (>90% agreement and kappa >0.84 for all items [32]) and the Food Environment Audit for Diverse Neighborhoods in 44 stores (89% of items had kappa >0.60) [31]; however, in these studies, surveyors received considerably more training than this study (2 days and 25 hours of training, respectively) [31,32]. The Store Scout app was designed to be used not only in research but also in practice to rapidly appraise the store environment and provide immediate feedback to retailers and recommendations for improvement. To replicate what was likely to be feasible in practice, we tested interrater reliability where surveyors received no or minimal training in the use of the tool.

We found that the scoring captured differences in the store environment across store types. Supermarkets, particularly large supermarkets, scored highest, indicating the most health-enabling store environments as assessed using the Store Scout app, whereas convenience and petrol stores scored lowest. This is consistent with previous research comparing supermarkets, petrol stations, and convenience stores [23,24,33], indicating construct validity. 

There is limited evidence comparing the food environment in remote versus nonremote supermarkets in Australia. Most research has focused on food prices, with food prices consistently higher in remote stores [16], whereas the Store Scout app takes a broader approach to food environment measurement and does not assess price other than price promotions. Cameron et al [34] assessed the shelf space and strategic placement of healthy and discretionary foods in urban, urban fringe, and rural or nonmetropolitan supermarkets in Victoria (from a single major Australian supermarket chain) and found that urban supermarkets had a generally healthier food environment compared with urban fringe and rural or nonmetropolitan stores; however, the urban fringe (most similar to the Brisbane sample in this study) and rural or nonmetropolitan supermarkets had similar healthy food environments overall. This is consistent with the findings in this study, but this is not a direct comparison, as the rural or nonmetropolitan centers in the Cameron study had populations >6000, more than double the largest remote community in the remote sample (median 740, range 220-2560).

### Strengths and Limitations

A strength of this study was that measurements were taken as would occur in practice (in physical stores during the day when the store is open) in several different store types and by surveyors who received no or minimal training in the use of the tool. A limitation is that in the remote sample, surveyors were not able to complete surveys simultaneously, which may have reduced interrater reliability, especially for meals and convenience foods as some ready-to-eat items (eg, pies, sandwiches, and salads) may only be available at certain times of the day.

Although we were able to examine internal consistency from up to 146 surveys in 2 very different retail contexts, we could not include all response combinations; however, there were still a median of 136 responses across the 35 measurement items used to assess internal consistency (range 63-146). The issues we experienced with missing data should not be an issue in future, as the app does not allow the surveyor to progress unless all measurement items are completed. The results reported here pertain mostly to surveys completed by surveyors with qualifications related to nutrition and/or public health. We cannot determine if the same degree of reliability and agreement would be achieved if this were not the case. We did not ask surveyors in the remote sample to record the time taken to record surveys; therefore, the median time of 25 min may not be applicable to this setting.

The difference when only practices related to unhealthy (or less healthy) products were considered is important. Approximately one-third of the energy in the Australian diet comes from discretionary (unhealthy) foods [35], and these foods are disproportionately represented on Australian food store shelves, and more likely to be promoted [36]. Although the Store Scout app assesses the store environment related to healthy and unhealthy (or less healthy) products, there are a greater number of measurement items related to healthy than unhealthy or less healthy products (156 versus 43, respectively), and practices discouraging purchases of unhealthy or less healthy products contribute only approximately 20% to the overall score. We are in the process of testing the criterion validity of the Store Scout mobile app against external measures (such as sales of healthy or unhealthy foods), and as part of this, we will investigate if higher weighting of practices related to unhealthy products leads to better validity of Store Scout scores.

### Conclusions

We developed a mobile app to rapidly appraise the in-store food retail environment across a wide range of healthy and unhealthy foods. We tested the Store Scout app in a diverse sample of 54 stores and found good to very good interrater reliability of measurement items and high internal consistency. We identified areas where tooltips and wording of measurement items can be improved to further increase the reliability of this tool. Scores reflecting how health enabling the store environment is are embedded in the app, giving the user a convenient mechanism for communicating results and initiating change with retailers or other stakeholders. We found high agreement between surveyors in overall scores and some category scores. This suggests that Store Scout overall scores are reliable for making comparisons across stores even when the tool is completed by different surveyors; however, category scores were less reliable. We found good distribution of scores and evidence that the scoring system can capture scoring by store type in line with previous evidence, indicating construct validity. The Store Scout mobile app shows promise in its ability to measure and track health-enabling store environments. In future projects, we will further assess the validity of this tool against external measures.

## Appendix

Multimedia Appendix 1 Numbers of surveys with complete and missing data.

Multimedia Appendix 2 Data included by analysis type.

Multimedia Appendix 3 Percentage agreement (%) between surveyors of Store Scout measurement items.

Multimedia Appendix 4 Internal consistency of Store Scout measurement items.

Multimedia Appendix 5 Kruskal Wallis equality-of-populations rank test results (*p* values).

Multimedia Appendix 6 Points for measurement items related to healthy products and unhealthy or less healthy products by store type.

## Abbreviations

AC: agreement coefficient
ALPA: Arnhem Land Progress Aboriginal Corporation
ICC: intraclass correlation coefficient
IRSAD: Index of Relative Socio-economic Advantage and Disadvantage
NHMRC: National Heart and Medical Research Council
