# Supplementary material for: A Mobile App to Rapidly Appraise the In-Store Food Environment: Reliability, Utility, and Construct Validity Study
Source: JMIR Mhealth Uhealth. 2020 Jul 22;8(7):e16971. doi: 10.2196/16971 (PMC7407248; doi:10.2196/16971)
Supplement: Multimedia Appendix 1 [file mhealth_v8i7e16971_app1.docx]

# Multimedia Appendix 1. Numbers of surveys with complete and missing data

| **Sample** | **All** | **Brisbane**  **Stage 1** | **Brisbane Stage 2** | **Healthy Stores 2020 Remote Stores** | | |
| --- | --- | --- | --- | --- | --- | --- |
|  |  |  |  | **Baseline** | **Intervention** | **Post-intervention** |
| Number of stores | 54^a^ | 17 | 17 | 20 | 20 | 20 |
| Total surveys collected | 148 | 34 | 34 | 20 | 40 | 20 |
| Surveys without data (all data missing) | 2 | 0 | 1 | 0 | 1 | 0 |
| Surveys with data | 146 | 34 | 33 | 20 | 39 | 20 |
| Complete data | 132 | 29 | 32 | 20 | 31 | 20 |
| Incomplete data | 16 | 5 | 1 | 0 | 8 | 0 |
| Missing data in one category only | 10 | 5 | 1 | 0 | 4 | 0 |
| Missing data in two categories only | 4 | 0 | 0 | 0 | 4 | 0 |

^a^ 54 unique stores; 94 unique data points by store and timepoint
