# Supplementary material for: A Mobile App to Rapidly Appraise the In-Store Food Environment: Reliability, Utility, and Construct Validity Study
Source: JMIR Mhealth Uhealth. 2020 Jul 22;8(7):e16971. doi: 10.2196/16971 (PMC7407248; doi:10.2196/16971)
Supplement: Multimedia Appendix 2 [file mhealth_v8i7e16971_app2.docx]

# Multimedia Appendix 2. Data included by analysis type

| **Analysis** | **Unit of analysis** | **Data type** | **Brisbane (Stage 1 & 2; n = 34 stores)** | **Remote stores (n = 20 stores)** | | |
| --- | --- | --- | --- | --- | --- | --- |
|  |  |  |  | **Baseline** | **Intv** | **Post** |
| 1. Inter-rater reliability | Paired | Items & Scores | n = 33 ^a^ | - | n = 19 ^a^ | - |
| 1. Internal consistency ^b^ | Survey | Items | n = 67 ^a^ | n = 20 | n = 39 ^a^ | n = 20 |
| 1. Utility of scoring | Store | Scores | n = 34 | n = 20 | - | - |
| 1. Construct validity (scores by type of store) | Store | Scores | n = 34 | n = 19 ^c^ | - | - |

- = Data not included; Intv = end of intervention period; Post = end of post-intervention period. Units of analysis are Survey (each survey included as one data point), Store (each store is included as one data point; average taken where there are values for more than one surveyor); Paired (paired surveys included as one data point;)

^a^ Survey data not available for one store (all data were missing for one surveyor), therefore this store/survey could not be included

^b^ Only some response combinations were assessable

^c^ Supermarkets only (petrol station excluded)
