# Supplementary material for: A Mobile App to Rapidly Appraise the In-Store Food Environment: Reliability, Utility, and Construct Validity Study
Source: JMIR Mhealth Uhealth. 2020 Jul 22;8(7):e16971. doi: 10.2196/16971 (PMC7407248; doi:10.2196/16971)
Supplement: Multimedia Appendix 3 [file mhealth_v8i7e16971_app3.docx]

# Multimedia Appendix 3. Percentage agreement (%) between surveyors of Store Scout measurement items

| **Category** | **All stores** | **Brisbane Stage 1** | **Brisbane Stage 2** | **Remote Stores** |
| --- | --- | --- | --- | --- |
| All | 83.2%; n=8584/10312 | 80.4%; n=2634/3278 | 87%; n=2764/3176 | 82.6%; n=3186/3858 |
| Breads & Cereals | 83.6%; n=853/1020 | 79.1%; n=253/320 | 85.6%; n=274/320 | 85.8%; n=326/380 |
| Dairy & Eggs | 86.7%; n=1228/1416 | 83.7%; n=364/435 | 92.2%; n=401/435 | 84.8%; n=463/546 |
| Drinks | 79.4%; n=1131/1425 | 73.9%; n=352/476 | 81%; n=363/448 | 83%; n=416/501 |
| Fruit & Vegetables | 84.5%; n=1315/1557 | 83.5%; n=426/510 | 89%; n=427/480 | 81.5%; n=462/567 |
| Meals & Convenience Foods | 83.6%; n=1468/1756 | 79.6%; n=417/524 | 88.5%; n=462/522 | 83%; n=589/710 |
| Meat & Seafood | 84%; n=900/1071 | 85.2%; n=304/357 | 86.3%; n=272/315 | 81.2%; n=324/399 |
| Snack Foods | 81.7%; n=1689/2067 | 79%; n=518/656 | 86.1%; n=565/656 | 80.3%; n=606/755 |
