# Supplementary material for: A Mobile App to Rapidly Appraise the In-Store Food Environment: Reliability, Utility, and Construct Validity Study
Source: JMIR Mhealth Uhealth. 2020 Jul 22;8(7):e16971. doi: 10.2196/16971 (PMC7407248; doi:10.2196/16971)
Supplement: Multimedia Appendix 4 [file mhealth_v8i7e16971_app4.docx]

# Multimedia Appendix 4. Internal consistency of Store Scout measurement items

| **Product type** | **Measurement items** | | **Internal consistency % (n)** | | | |
| --- | --- | --- | --- | --- | --- | --- |
|  | **Higher order** | **More Specific** | **All** | **Brisbane Stage 1** | **Brisbane Stage 2** | **Remote Stores** |
| Fruit/vegetables | Price promotion | Is the price promotion visible/easy to see? | 100%  (n=146/146) | 100%  (n=33/33) | 100%  (n=33/33) | 100%  (n=80/80) |
| Fruit/vegetables | Promotion | Does the promotion stand out? | 100%  (n=134/134) | 100%  (n=34/34) | 100%  (n=33/33) | 100%  (n=67/67) |
| Bottled water | Product availability | Visible on entering store | 99.2%  (n=118/119) | 96.2%  (n=25/26) | 100%  (n=17/17) | 100%  (n=76/76) |
| Bottled water | Product availability | At front of store | 100%  (n=126/126) | 100%  (n=27/27) | 100%  (n=23/23) | 100%  (n=76/76) |
| Bottled water | Product availability | In all drinks fridges | 98.4%  (n=62/63) | 96.8%  (n=30/31) | 100%  (n=16/16) | 100%  (n=16/16) |
| Bottled water | Product availability | Easy to find | 99.3%  (n=139/140) | 96.8%  (n=30/31) | 100%  (n=30/30) | 100%  (n=79/79) |
| Refrigerated water | Product availability | Refrigerated water at child eye level/arm’s reach | 97.9%  (n=139/142) | 93.8%  (n=30/32) | 96.9%  (n=31/32) | 100%  (n=78/78) |
| Diet/NAS cordial | Product availability | Diet/NAS option easy to find | 97.6%  (n=124/127) | 93.8%  (n=30/32) | 100%  (n=33/33) | 98.4%  (n=61/62) |
| Diet/NAS cordial | Product availability | Diet/NAS option near full sugar option | 97.9%  (n=139/142) | 93.9%  (n=31/33) | 100%  (n=33/33) | 98.7%  (n=75/76) |
| Diet/NAS cordial | Product availability | Diet/NAS option same or more space as full sugar cordial | 97.6%  (n=82/84) | 93.5%  (n=29/31) | 100%  (n=22/22) | 100%  (n=31/31) |
| Water or diet drinks | Price promotion | Is the price promotion visible/easy to see? | 100%  (n=140/140) | 100%  (n=32/32) | 100%  (n=33/33) | 100%  (n=75/75) |
| Water or diet drinks | Promotion | Does the promotion stand out? | 100%  (n=134/134) | 100%  (n=32/32) | 100%  (n=32/32) | 100%  (n=70/70) |
| Healthier sweet biscuits & cakes | Product availability | Healthier option easy to find | 95.9%  (n=117/122) | 89.3%  (n=25/28) | 95.8%  (n=23/24) | 98.6%  (n=69/70) |
| Healthier savoury biscuits | Product availability | Healthier option easy to find | 98.4%  (n=120/122) | 96%  (n=24/25) | 96.7%  (n=29/30) | 100%  (n=67/67) |
| Healthier savoury biscuits | Product availability | Healthier option near regular option | 97.9%  (n=141/144) | 97%  (n=32/33) | 97%  (n=32/33) | 98.7%  (n=77/78) |
| Healthier savoury biscuits | Product availability | Healthier option at eye level/arm's reach | 97.6%  (n=123/126) | 96.8%  (n=30/31) | 97%  (n=32/33) | 98.4%  (n=61/62) |
| Healthier snack foods | Price promotion | Is the price promotion visible/easy to see? | 100%  (n=145/145) | 100%  (n=33/33) | 100%  (n=33/33) | 100%  (n=79/79) |
| Healthier snack foods | Promotion | Does the promotion stand out? | 100%  (n=144/144) | 100%  (n=33/33) | 100%  (n=33/33) | 100%  (n=78/78) |
| Healthier meals/convenience foods | Product availability | Is the price promotion visible/easy to see? | 99.3%  (n=141/142) | 100%  (n=32/32) | 100%  (n=33/33) | 98.7%  (n=76/77) |
| Healthier meals/convenience foods | Product availability | Does the promotion stand out? | 100%  (n=143/143) | 100%  (n=33/33) | 100%  (n=32/32) | 100%  (n=78/78) |
| Wholemeal/multigrain bread | Product availability | Wholemeal/multigrain easy to find | 100%  (n=133/133) | 100%  (n=32/32) | 100%  (n=33/33) | 100%  (n=68/68) |
| Wholemeal/multigrain bread | Product availability | Wholemeal/multigrain at eye level/arm's reach | 100%  (n=126/126) | 100%  (n=33/33) | 100%  (n=33/33) | 100%  (n=60/60) |
| Wholemeal/multigrain bread | Product availability | Wholemeal/multigrain same or more space as regular white bread | 100%  (n=81/81) | 100%  (n=30/30) | 100%  (n=16/16) | 100%  (n=35/35) |
| Healthier breads & cereals | Price promotion | Is the price promotion visible/easy to see? | 100%  (n=143/143) | 100%  (n=32/32) | 100%  (n=31/31) | 100%  (n=80/80) |
| Healthier breads & cereals | Promotion | Does the promotion stand out? | 100%  (n=144/144) | 100%  (n=33/33) | 100%  (n=33/33) | 100%  (n=78/78) |
| Healthier meat & seafood | Price promotion | Is the price promotion visible/easy to see? | 99.3%  (n=144/145) | 97.1%  (n=33/34) | 100%  (n=32/32) | 100%  (n=79/79) |
| Healthier meat & seafood | Promotion | Does the promotion stand out? | 100%  (n=140/140) | 100%  (n=34/34) | 100%  (n=32/32) | 100%  (n=74/74) |
| Cheese | Product availability | Easy to find | 98.5%  (n=135/137) | 100%  (n=29/29) | 96.8%  (n=30/31) | 98.7%  (n=76/77) |
| Cheese | Product availability | At eye level/arms reach | 98.6%  (n=138/140) | 100%  (n=32/32) | 96.9%  (n=31/32) | 98.7%  (n=75/76) |
| Cheese | Product availability | Well-presented | 98.5%  (n=134/136) | 100%  (n=31/31) | 96.9%  (n=31/32) | 98.6%  (n=72/73) |
| Fresh eggs | Product availability | Easy to find | 99.2%  (n=122/123) | 96.2%  (n=25/26) | 100%  (n=30/30) | 100%  (n=67/67) |
| Fresh eggs | Product availability | At eye level/arms reach | 99%  (n=103/104) | 96.7%  (n=29/30) | 100%  (n=31/31) | 100%  (n=43/43) |
| Fresh eggs | Product availability | Well-presented | 99.3%  (n=135/136) | 96.8%  (n=30/31) | 100%  (n=31/31) | 100%  (n=74/74) |
| Healthier dairy & eggs | Price promotion | Is the price promotion visible/easy to see? | 100%  (n=141/141) | 100%  (n=31/31) | 100%  (n=31/31) | 100%  (n=79/79) |
| Healthier dairy & eggs | Promotion | Does the promotion stand out? | 100%  (n=135/135) | 100%  (n=30/30) | 100%  (n=30/30) | 100%  (n=75/75) |

Internal consistency calculated as surveys with consistent responses as a percentage of all surveys where consistency could be assessed (ie: excluding non-assessable responses) where: *Consistent responses =* “yes” or “no” for both measurement items; *Inconsistent response*s = “no” to higher order measurement item and “yes” to more specific item; *Non-assessable responses =* “yes” to higher order item and “no” to more specific item.
