# Supplementary material for: A Mobile App to Rapidly Appraise the In-Store Food Environment: Reliability, Utility, and Construct Validity Study
Source: JMIR Mhealth Uhealth. 2020 Jul 22;8(7):e16971. doi: 10.2196/16971 (PMC7407248; doi:10.2196/16971)
Supplement: Multimedia Appendix 5 [file mhealth_v8i7e16971_app5.docx]

# Multimedia Appendix 5. Kruskal Wallis equality-of-populations rank test results (p values)

|  | Petrol Stations | Convenience stores | Large Supermarkets | Small Supermarkets |
| --- | --- | --- | --- | --- |
| Convenience stores | 0.2754 |  |  |  |
| Large Supermarkets | **<0.0001*** | **<0.0001*** |  |  |
| Small Supermarkets | **0.0064** | **0.0064** | **0.0068** |  |
| Remote supermarkets | **0.0021*** | **0.0009*** | **0.0032*** | 0.4269 |

* Denotes relationships remaining significant after Holm-Bonferroni adjustment for multiple comparisons
