# Supplementary material for: A Mobile App to Rapidly Appraise the In-Store Food Environment: Reliability, Utility, and Construct Validity Study
Source: JMIR Mhealth Uhealth. 2020 Jul 22;8(7):e16971. doi: 10.2196/16971 (PMC7407248; doi:10.2196/16971)
Supplement: Multimedia Appendix 6 [file mhealth_v8i7e16971_app6.docx]

# Multimedia Appendix 6. Points for measurement items related to healthy products and unhealthy or less healthy products by store type

Higher points indicate the store is more in line with best practice. *Healthy products points* relate to healthy products being available, placed prominently or promoted. *Unhealthy product points* relate to unhealthy or less healthy products not being available, placed prominently or promoted. Some measurement items are equally related to healthy and unhealthy or less healthy products (eg same or more space for healthy versus less healthy). These have been included in the unhealthy product points, however results are similar if these questions are excluded.
